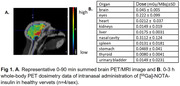# First Comprehensive Intranasal PET imaging of Insulin using the Aptar device in Nonhuman Primates

**DOI:** 10.1002/alz.091073

**Published:** 2025-01-09

**Authors:** Kiran K. Solingapuram Sai, Krishna Kumar Gollepalli, Jennifer M. Erichsen, Ivan Krizan, Mack Miller, Matthew J. Jorgensen, Suzanne Craft

**Affiliations:** ^1^ Wake Forest University School of Medicine, Winston‐Salem, NC USA

## Abstract

**Background:**

Insulin resistance (IR) is associated with abnormal tau‐phosphorylation and IR markers in AD brain co‐localize with neurofibrillary tangles. One strategy to overcome brain IR is to increase brain insulin is via intranasal insulin (INI) administration using specialized intranasal devices that deliver insulin to the brain. Our recent INI vs. placebo‐controlled double‐blinded clinical trial in MCI/AD showed that INI improved CSF biomarkers and slowed symptom progression over the placebo group treated with one type of delivery device (with no benefit with another). While these studies highlight the potential of INI for therapeutic applications, methods are needed to verify the successful INI delivery with specific devices. Here we report our novel [^68^Ga]‐NOTA‐insulin‐based brain PET imaging and whole‐body dosimetry through intranasal route in healthy vervets using the Aptar Cartridge Pump System (CPS) device for the first time.

**Method:**

Human‐grade insulin was chelated with NOTA and radiolabeled with [^68^Ga]. 0‐90min and 0‐3h PET scans were conducted for brain and whole‐body dosimetry in healthy vervets (n=4/sex, 9‐12 y) after intranasal administration (2 puffs per nostril) of [^68^Ga]‐NOTA‐insulin (25‐37 MBq) through the Aptar device. Blood glucose, oxygen, heartbeat, and temperature were collected throughout the scan. Standard uptake values (SUVs), time‐activity curves (TACs), and standard dosimetry organ doses were calculated.

**Result:**

[^68^Ga]‐NOTA‐insulin radiochemistry was fully‐optimized: shorter reaction times (∼6‐7min), lower insulin mass (2‐4 units), high radiochemical purity (>99%), and molar activity (108 GBq/µmol). No significant changes were observed in vitals recorded. PET/MRI images showed brain penetration; SUV whole‐brain=0.29±0.11, hippocampus=0.18±0.05, cortex=0.21±0.08, choroid plexus=0.38±0.11, and cerebellum=0.09±0.01. TACs showed that radioactivity peaked in brain within 15‐20min of radiotracer administration and washed out by 90 min. Dosimetry showed nasal cavity and eyes as critical organs and effective dose=0.11 mSv/MBq. No sex differences were seen in brain uptake or dosimetry parameters.

**Conclusion:**

Brain PET and whole‐body dosimetry PET scans of [^68^Ga]‐NOTA‐insulin in vervets demonstrate brain penetration and favorable distribution of INI respectively. This preliminary data provides critical information for translating [^68^Ga]‐NOTA‐insulin PET with intranasal delivery to humans using the Aptar device. This strategy will create a new paradigm for understanding the INI in AD pathogenesis and advance novel therapeutic platforms.